# Antibacterial Activity of a Natural Clay Mineral against *Burkholderia cepacia* Complex and Other Bacterial Pathogens Isolated from People with Cystic Fibrosis

**DOI:** 10.3390/microorganisms11010150

**Published:** 2023-01-06

**Authors:** Shekooh Behroozian, James E. A. Zlosnik, Wanjing Xu, Loretta Y. Li, Julian E. Davies

**Affiliations:** 1 Department of Chemical and Biological Engineering, University of British Columbia, 2360 E Mall, Vancouver, BC V6T 1Z3, Canada; 2 Centre for Understanding and Preventing Infection in Children, Division of Infectious Diseases, Department of Pediatrics, BC Children’s Hospital Research Institute, University of British Columbia, Vancouver, BC V5Z 4H4, Canada; 3 Department of Civil Engineering, University of British Columbia, 6250 Applied Science Ln, Vancouver, BC V6T 1Z3, Canada; 4 Department of Microbiology and Immunology, University of British Columbia, 2350 Health Sciences Mall, Vancouver, BC V6T 1Z3, Canada

**Keywords:** natural clay mineral, cystic fibrosis, antibacterial activity, *Burkholderia cepacia* complex (Bcc), *Pseudomonas aeruginosa*, *Stenotrophomonas maltophilia*, chronic lung infections

## Abstract

There is an impending crisis in healthcare brought about by a new era of untreatable infections caused by bacteria resistant to all available antibiotics. Thus, there is an urgent need to identify novel antimicrobial agents to counter the continuing threat posed by formerly treatable infections. We previously reported that a natural mineral clay known as Kisameet clay (KC) is a potent inhibitor of the organisms responsible for acute infections. Chronic bacterial infections present another major challenge to treatment by antimicrobials, due to their prolonged nature, which results in repeated exposure to antibiotics and a constant selection for antimicrobial resistance. A prime example is bacteria belonging to the *Burkholderia cepacia* complex (Bcc), which particularly causes some of the most serious chronic lung infections in patients with cystic fibrosis (CF) associated with unpredictable clinical outcomes, poor prognosis, and high mortality rates. Eradication of these organisms from CF patients with limited effective antimicrobial options is a major challenge. Novel therapeutic approaches are urgently required. Here, we report the in vitro antibacterial activity of KC aqueous suspensions (1–10% *w*/*v*) and its aqueous extract (L100) against a collection of extensively and multi-drug resistant clinical isolates of Bcc, *Pseudomonas aeruginosa*, and *Stenotrophomonas maltophilia* isolated from patients with CF. These findings present a potential novel therapy for further investigation in the clinic.

## 1. Introduction

Recalcitrant chronic bacterial infections in humans represent a significant therapeutic problem worldwide whereby repeated challenges with antibiotics promote the acquisition of drug-resistant bacteria by constant selection for new infections with intrinsically resistant organisms and selective pressures on existing organisms, further elevating their resistance [1]. This situation is intensified by the rapid spread of resistance genes, particularly among Gram-negative bacteria [2]. Cystic fibrosis (CF) is one such condition that renders affected individuals susceptible throughout their lives to chronic and ultimately deteriorating multi-year lung infections, which account for over 90% of the morbidity and mortality associated with the disease [1,3,4,5,6]. Originally described in 1938 [7], CF is a life-limiting inherited multi-organ disease due to one of over 2000 gene mutations of cystic fibrosis transmembrane conductance regulator (CFTR) gene leading to the dysfunction or absence of the CFTR protein, a membrane anion channel that regulates the transepithelial ion flow vital to maintaining the proper ion and water transport and epithelial surface hydration [4,8,9,10,11]. Abnormally viscous secretions in organ systems containing epithelial -most crucially in the lungs, pancreas, liver, and gastrointestinal tract- cause obstructions that lead to further inflammation and tissue damage [12,13]. Reportedly, dysfunctional or absent CFTR causes a wide disease spectrum in people with CF (pwCF) among which chronic endobronchial infections and exocrine pancreatic insufficiency remain the main clinical manifestation of the disease [9,14,15]. Although earlier publications emphasize that CF was typical in Caucasian populations by primarily affecting those of European descent, increasing worldwide awareness and reports illustrate a changing demography in which CF is not an uncommon genetic disorder in other races and ethnicities around the globe [11,16].

It has long been demonstrated that pwCF are prone to complex, polymicrobial pulmonary infections caused by a range of multi-drug resistant (MDR) opportunistic Gram-negative bacteria including *Pseudomonas aeruginosa*, *Burkholderia cepacia* complex (Bcc), *Achromobacter* species, and *Stenotrophomonas maltophilia*, among which Bcc members are particularly the most threatening and virulent pathogens isolated from pwCF [17,18,19,20,21].

The Bcc group comprises at least 20 phenotypically similar, phylogenetically closely related, diverse, and highly adaptable bacterial species [20,21]. Prior to the 1990s, Bcc was known as one species, *Burkholderia cepacia* [22]. Initially, *B. cepacia* was named *Pseudomonas cepacia* when first isolated from pwCF in 1977 [23]. It took until the mid-1990s when researchers found that *B. cepacia* was, indeed, composed of multiple distinct subgroups, and initially, five genomovars were identified as *B. cepacia* (genomovar I), *B. multivorans* (II), *B. cenocepacia* (III), *B. stabilis* (IV), *B. vietnamiensis* (V) [24]. The Bcc bacteria have emerged since the 1980s [25] as highly problematic opportunistic pathogens in immunocompromised individuals, most notably among pwCF, as well as patients who suffer from chronic granulomatous disease (CGD) [20,22,26,27,28,29,30].

Persistent pulmonary infections caused by Bcc bacteria remain a threat to pwCF due to their unpredictable infection trajectory ranging from a chronic asymptomatic phenotype to an uncertain, rapid fulminant respiratory failure, and septicaemic fatal cepacia syndrome [30,31,32]. These infections are associated with unforeseeable rates of progressive decline in lung function, poor prognosis, prolonged hospitalization, and elevated morbidity and mortality rates, especially among patients with more advanced pulmonary exacerbation or lung transplantation [28,31,33]. In fact, many transplantation centers refuse CF patients harboring Bcc as these pathogens cause death and disparity in post-transplant outcomes through immediate invasive disease and cepacia syndrome [34,35]. Outbreaks of different Bcc members are often reported, and a large body of evidence indicates their spread in a patient-to-patient manner [36].

The pathogenic mechanisms employed by Bcc members in pwCF are not fully understood but are likely due to multiple factors including high levels of both intrinsic and acquired mechanisms of resistance to diverse classes of antimicrobial agents [37,38]. Bcc species, particularly *B. cenocepacia*, exhibit heterogeneous resistance to a broad range of antibacterial agents due to periplasmic or membrane-bound β-lactamases, efflux pump-mediated MDR, restrictive porins, outer membrane (OM) permeability barriers, and alteration in drug targets [38,39,40]. Their ability to evade the inhibitory action of multiple classes of antibiotics together with their in vivo biofilm formation account for the serious nature of persistent infections and extreme difficulty in their elimination [40,41,42,43,44]. Moreover, the known high risk of inter-patient transmissibility is associated with adverse clinical courses, ranging from mild asymptomatic carriage to a fulminant decline in pulmonary function, and cepacia syndrome [29,30,32,41]. Noteworthy, these characteristics coupled with their high adaptability to environmental changes, make Bcc infections extremely challenging [40]. As there are few effective treatments for these deadly pathogens, novel therapeutic strategies are urgently needed [38,40,45].

Given the scientific and economic challenges facing the discovery of novel antimicrobial agents, relatively few active candidates are currently being developed in the pipeline [46,47]. Thus, exploring untapped natural sources may yield novel therapies in the battle against the escalating emergence of untreatable MDR pathogens [2,33]. Complementary and alternative medicines which exhibit antimicrobial properties could address this issue [47]. Recently, “historical” agents such as natural clay minerals with demonstrated curative applications and reported antibacterial activities have been of increasing interest [48,49].

Extending back to prehistory, natural clay minerals have been used by humans for medicinal, nutritional, and protective purposes [48,50,51,52]. Ancient evidence illustrates their successful applications for healing wounds, alleviating irritations, and cleaning skin (as anti-inflammatory or antiseptic agents) [48]. Clay minerals are the most abundant chemically-active constituents of the Earth’s surface with a defined nanostructure of geological origin [50,53]. They consist of mainly microcrystalline particles of hydrous charged sheet silicates (phyllosilicates) and of aluminum or magnesium silicates [52,54]. They possess specific physicochemical properties such as ultra-fine grain size (one dimension < 2.0 μm), vast specific surface area (~100′s m^2^/g), and ion exchange capacity via the intercalation of ions and retaining them in an exchangeable state [54,55].

The potential of natural clay minerals as antimicrobial agents received specific attention when, recently, a successful application of hydrated French green clay poultices for the treatment of advanced Buruli ulcer, caused by a pathogenic bacterium *Mycobacterium ulcerans*, was reported [56]. This prompted interest in investigating the physicochemical features and antibacterial properties of clay minerals in vitro [57,58,59]. A decade of studies since then has revealed that few deposits among natural healing clays worldwide exhibited antibacterial properties [60,61]. Notably, despite their different mineralogical and physicochemical characteristics, they all originated from hydrothermally altered volcanic clastic environments, containing nanoscale expandable clay minerals and iron-rich phases, and generating pH levels of either <5 or >10 by hydration [59,61].

Kisameet clay (KC), a naturally occurring glacial clay mineral from Kisameet Bay on the central coast of British Columbia, Canada [62], has been long recognized as a healing clay by the local Heiltsuk First Nations people due to its exceptional curative properties for treating various types of skin irritation and internal maladies [62,63]. In the 1940s KC aqueous suspensions were sold as a natural remedy for topical or oral administrations and numerous anecdotal reports suggest the effective therapeutic application of KC against diverse ailments, including topical applications for wounds, burns, arthritis, and skin irritation, or by oral consumption of clay suspensions for treating internal maladies such as duodenal ulcer and ulcerative colitis [63]. Despite those observations, there was limited experimental information concerning the biological, and physicochemical composition of KC related to its antimicrobial properties and spectrum of activity [62,63].

We recently performed a series of integrated studies of microbiological, mineralogical, and physicochemical properties of KC samples collected from different sites at the deposit on Kisameet Bay [64,65]. Our studies provided a better insight into what might make this clay active and highlighted the diversity and complexity of the KC deposit [64]. Moreover, we showed that KC is the first natural clay mineral found to be active against two major fungal pathogens (*Candida albicans* and *Cryptococcus neoformans*), as well as bacterial biofilms in vitro [65,66]. Through further investigation aiming to unravel the mechanism(s) of its antibacterial properties, we found that the broad-spectrum antibacterial features of KC can be extracted in water (aqueous leachates) [65,66].

Our previous studies demonstrated that aqueous suspensions of KC have a potent broad-spectrum antibacterial activity in vitro against the major nosocomial pathogens, the ESKAPE organisms (*Enterococcus faecium*, *Staphylococcus aureus*, *Klebsiella pneumoniae*, *Acinetobacter baumannii*, *P. aeruginosa*, and *Enterobacter* species) responsible for acute infections in hospitals [67]. Here, to investigate whether KC and its aqueous extract may have value in the treatment of the range of serious bacterial pathogens involved in chronic pulmonary infections in CF patients, we conducted a study with a collection of clinical isolates of Bcc, *P. aeruginosa*, and *S. maltophilia* collected from pwCF.

## 2. Materials and Methods

### 2.1. Bacterial Strains and Growth Conditions

This study was conducted on 17 clinical isolates consisting of twelve Bcc, four *P. aeruginosa*, and one *S. maltophilia* deposited at the Canadian *Burkholderia cepacia* Complex Research and Referral Repository (CBCCRRR) at the University of British Columbia, Vancouver, Canada. All the bacteria were isolated from collected clinical specimens from the respiratory tract (either sputum, throat, or cough swabs) or the blood of pwCF attending pediatric or adult CF clinics in Vancouver, BC between 1990 and 2015. Initial identification of bacterial isolates was carried out in the diagnostic microbiology labs at those clinics. Isolates were then transferred to CBCCRRR for identification at the species level as described previously [41]. Briefly, this was performed through a polyphasic approach using both phenotypic and genetic assays, *recA* polymerase chain reaction (PCR) analysis, and sequencing of this multilocus sequence typing allele [41]. Typing at the strain level was carried out using random amplified polymorphic DNA (RAPD) analysis [41]. All the isolates were then frozen and stored before experimental testing. The Bcc isolates include six *B. cenocepacia*, two *B. multivorans*, and one of each *B. cepacia*, *B. stabilis*, *B. dolosa*, and *B. vientnamiensis*. Among Bcc isolates, *B. cenocepacia* and *B. multivorans* strains were sequential isolates collected from three patients at different times. Out of four *P. aeruginosa* isolates, VC8263 and VC17829 were epidemic Type strains of A002 and A097, respectively [68,69]. All isolates were grown in Lysogeny broth (LB) (Miller) or on LB agar at 37 °C.

### 2.2. Clay Mineral Sample

The unprocessed natural Kisameet clay (KC-35) mineral used in this investigation was supplied by Kisameet Glacial Clay Inc. (West Vancouver, BC, Canada) in its original wet form. Following transport to the University of British Columbia, the clay sample was stored and sealed at 4 °C under normal atmospheric conditions in the dark. This clay sample previously exhibited potent antibacterial activity [67]. The clay sample was dried in a vacuum desiccator at room temperature. Dry KC samples were ground using a mortar and pestle, autoclaved at 121 °C for 1 h, and stored at room temperature before experimental testing. Measurement of pH was performed on equilibrated suspensions of 1 g KC mineral in 10 mL deionized water (dH_2_O) or aqueous leachate using a VWR-SB20 pH meter.

### 2.3. Antimicrobial Susceptibility Assay

Antimicrobial resistance profiles of isolates were characterized by standard agar disk diffusion susceptibility assays based on the Bauer-Kirby method and updated protocol provided by the American Society for Microbiology [70,71]. Susceptibility assays were carried out using cation-adjusted Mueller Hinton-II (MH) broth and agar media and a panel of 34 antibiotic disks (Oxoid, BBL) representing antibacterial agents from 14 different classes. In brief, an overnight culture of each isolate in MH broth was diluted and incubated with gentle shaking to reach the mid-exponential phase of growth. Then, MH agar plates were inoculated with the bacterial cultures and antibiotic disks were placed on the inoculated plates and incubated at 37 °C for 20–24 h before the zones of inhibition (ZOI) were measured. Experiments were performed at least three times.

### 2.4. Preparation of Aqueous Suspensions and Leachate of Clay

Aqueous suspensions of clay with 1 or 10% KC (*w*/*v*) concentrations were prepared by suspending 10 or 100 mg of dry, ground, autoclaved clay in 1 mL of sterile dH_2_O, respectively. To study the soluble fraction of KC suspensions, aqueous leachate (L100) preparations were first obtained by adding 2 g of autoclaved KC to 20 mL of sterile dH_2_O resulting in 10% (*w*/*v*) aqueous suspensions. After continuous stirring for 24 h at room temperature, KC suspensions were then centrifuged at 25,000 revolutions per minute (rpm) for 2 h at 4 °C to separate insoluble minerals. The supernatants were sterilized and clarified by passage through 0.22 μm (Millipore) filters. The obtained aqueous leachate (L100) was the sterile, clear, soluble fraction of KC suspensions after the removal of clay particles through ultracentrifugation and filter sterilization.

### 2.5. Antibacterial Assay with Clay Suspensions or Aqueous Leachates

An in vitro assay was used to examine the effect of KC on these bacterial isolates as described previously [67]. Briefly, overnight cultures of bacteria were diluted into the fresh LB broth to an approximate concentration of ~10^7^ colony forming units (CFU) mL^−1^ and incubated at 37 °C with gentle mixing on an orbital rotating platform (200 rpm) to reach the mid-logarithmic phase of growth. Aqueous suspensions of 1 or 10% KC (*w*/*v*) were prepared by suspending dried, ground, autoclaved clay in sterile dH_2_O. Bacterial cells were collected by centrifugation, rinsed once with sterile phosphate-buffered saline (PBS pH 7.4), and re-suspended at ~10^7^ CFU mL^−1^ in either 1% (*w*/*v*) KC suspensions (for *P. aeruginosa* and *S. maltophilia* isolates), or 10% (for all Bcc isolates), or in dH_2_O (as the viability control in the absence of KC). Then, suspensions were incubated with gentle shaking (200 rpm) at 37 °C to provide proper contact with clay particles and prevent sedimentation. Bacterial viability was determined using 10-fold serial dilution plating of aliquots removed at the start of experiments (time 0) and after 5, 24, and 48 h following exposure to clay suspensions. Antibacterial assays with KC leachate (L100) were performed similarly. The washed pellets of bacterial isolates were resuspended at ~10^7^ CFU mL^−1^ in KC leachate samples and incubated at the same condition as described above. Viability counts were performed by removing aliquots at the start of the experiment and three time points within 48 h of exposure to L100.

## 3. Results

### 3.1. All the CF Isolates Exhibited Extensively Drug-Resistant (XDR) or MDR Profiles

Antimicrobial resistance profiles of isolates showed that all the *B. cenocepacia*, *B. cepacia*, and *B. stabilis* isolates presented XDR phenotypes [72]; widespread MDR was observed among the other isolates (Table 1). All isolates exhibited resistance to first- and second-generation cephalosporins, ertapenem, meropenem, amoxicillin-clavulanic acid, ampicillin, and nitrofurantoin. In addition, all *B. cenocepacia*, *B. cepacia*, *B. multivorans* VC5602, and *S. maltophilia* isolates were resistant to all six aminoglycosides and also spectinomycin tested, while *P. aeruginosa* strains were resistant to cefixime, cefpodoxime, sulfamethoxazole-trimethoprim, and trimethoprim. Moreover, resistance to sulfonamides, tetracycline, and trimethoprim was observed among all six *B. cenocepacia* isolates and *B. cepacia* strain. All the Bcc isolates, except for *B. cenocepacia* C9343 and *B. stabilis*, exhibited resistance to polypeptides (colistin and polymyxin B). Collectively, a few isolates were resistant to ceftazidime and piperacillin. Furthermore, sequential isolates of *B. cenocepacia* and *B. multivorans* showed some differences in their resistance profiles.

### 3.2. KC Aqueous Suspensions and Leachate Showed Potent Antibacterial Activity against All the Isolates

**Figure 1:**
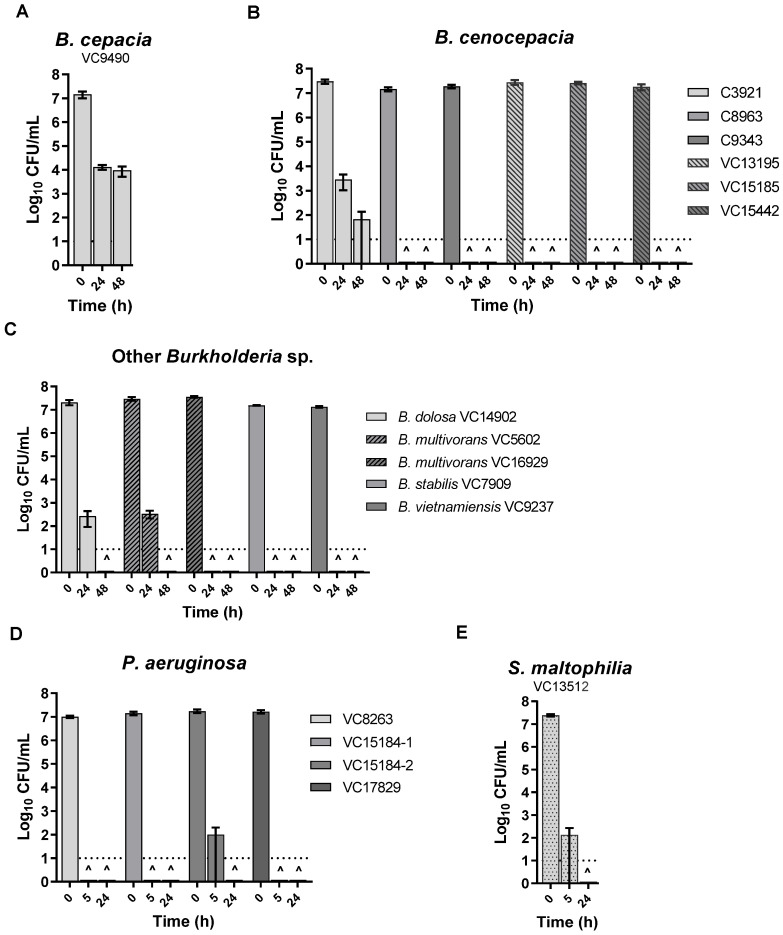
Effect of aqueous suspensions of KC on the viability of isolates: 10% (*w*/*v*) against *B. cepacia* complex isolates (**A**–**C**) and 1% (*w*/*v*) against *P. aeruginosa* isolates (**D**) and *S. maltophilia* isolate (**E**). CFUs have been determined at 0 h, 5 h, 24 h, and 48 h of incubation. **^** indicates that no viable cell could be recovered at that time point. Error bars represent the standard error (SE) of the mean of three independent replicates of each strain. The dotted line at log_10_ = 1 of the *Y* axis represents the limit of detection for CFUs.

To examine the effect of KC on the isolates, an in vitro assay using 1 or 10% (*w*/*v*) aqueous suspensions of KC (pH 4.3–4.5) was performed as described previously [67]. As shown in Figure 1 exposure to KC reduced, and in most cases eliminated, the viability of all isolates tested. After 24 h treatment of Bcc strains with 10% (*w*/*v*) KC suspensions, no viable cells could be recovered except for *B. dolosa* and *B. multivorans* VC5602, which required up to 48 h (Figure 1B,C) and *B. cepacia* and *B. cenocepacia* C3921 that showed a 3–5 log_10_ decline in CFUs in the same period of treatment (Figure 1A,B). A 1% (*w*/*v*) suspension of KC did not inhibit Bcc isolates (data not shown) but caused a loss of the viability of *P. aeruginosa* VC8263, VC15184-1, and VC17829 in 5 h and *P. aeruginosa* VC15184-2 and *S. maltophilia* within 24 h of treatment (Figure 1D,E). In contrast, the viability controls without KC exhibited less than one log_10_ decline in CFU counts during the same periods of incubation (data not shown).

**Figure 2:**
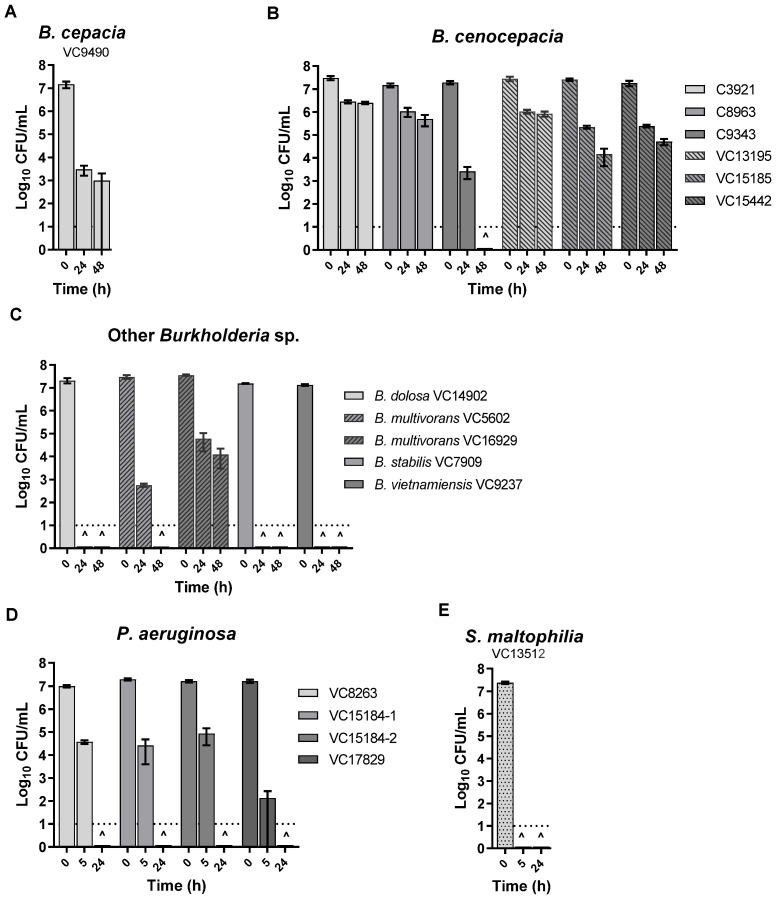
Effect of aqueous leachate of KC (L100) on the viability of *B. cepacia* complex isolates (**A**–**C**), *P. aeruginosa* isolates (**D**), and *S. maltophilia* isolate (**E**). CFUs have been determined at 0 h, 5 h, 24 h, and 48 h of incubation. **^** indicates that no viable cell could be recovered at that time point. Error bars represent the standard error (SE) of the mean of three independent replicates of each strain. The dotted line at log_10_ = 1 of the *Y* axis represents the limit of detection for CFUs.

Here, to test if the antibacterial effect was due to soluble components released from KC particles, a water-leachable fraction of the KC suspension was prepared and assayed for activity against these isolates. Figure 2 illustrates that the KC aqueous leachate (L100, pH 3.9) was bactericidal to most of the isolates. While *B. dolosa*, *B. stabilis*, *B. vietnamiensis*, all *P. aeruginosa*, and *S. maltophilia* strains exhibited loss of viability after 24 h of treatment with L100 (Figure 2C–E), the same bactericidal effect took 48 h for *B. cenocepacia* C9343 and *B. multivorans* VC5602 (Figure 2B,C). *B. cepacia* exhibited more than a 4 log_10_ decline in CFU during the period of treatment and all *B. cenocepacia* strains, other than C9343, and also *B. multivorans* VC16929 showed a ~1–3 log_10_ decline in the viability (Figure 2A–C).

## 4. Discussion

CF once known as an untreatable uniformly fatal disease in early childhood among the Caucasian population is now recognized as a globally distributed disease of a new face with prolonged survival to adulthood [11,33]. Over six decades, there has been a remarkable improvement in health outcomes as well as a substantial increase in the life expectancy of pwCF [33]. The basis for the improvement in clinical and health outcomes is multifactorial, much of which is associated with advancements in antimicrobial treatments [45]. Despite such substantial advances, CF-associated morbidity is still dominated by recurrent pulmonary infections as the most severe manifestation of the disease [3,11,33]. Thus, continued development and optimal application of novel antimicrobial agents (including antibiotic combinations) are vital to improving the survival and life quality of pwCF. As the hallmark of CF pulmonary disease, an early and persistent bacterial infection is the major determinant of life span in affected patients [3,15,44]. The most challenging problem in the management of CF is the early development of chronic infections, which requires successful colonization followed by long-term organism survival and, typically, additional antimicrobial resistance [15,16]. In spite of continuous changes in the epidemiology of CF pathogens, Bcc still remains the most feared threat to pwCF [30,32].

The Bcc species of bacteria are responsible for the most challenging of all pulmonary infections in pwCF due to their remarkable resistance to most available therapeutic agents making them virtually difficult to treat [28,32,40,41]. As current antimicrobial options for Bcc are limited and antimicrobial resistance evolves rapidly, the development of novel therapeutic strategies aimed at disarming Bcc bacteria and other MDR infections from pwCF needs ongoing investigation [38,39]. While Bcc-infected CF patients are in dire need of effective therapeutics, some studies have bridged the gap by exploring novel approaches including antibiotic combination therapies [79,80,81], screening natural antimicrobial compounds such as medical plant-derived small molecules [82,83], and novel aerosolized antibiotic formulations [45,84,85]. One of the most significant advances in CF therapeutics has been the development of CFTR modulators, (both potentiators and correctors) as a corrective strategy, while bacteriophage therapy, vaccine strategies, and immunotherapy remain mostly experimental [3,80,86,87,88].

This study demonstrates the in vitro antibacterial effect of KC and its aqueous leachate (L100) against all the clinical isolates tested, including sequential isolates from chronic infections of *B. cenocepacia* and *B. multivorans*, the two most common Bcc species that account for around 85–97% of all Bcc infections [36,38,89], and *P. aeruginosa* isolates as the most common pathogen in pwCF [17,75]. These data confirmed our previous observations on the potent bactericidal effect of KC against MDR clinical isolates of *P. aeruginosa* [67]. Notably, two epidemic Type strains of *P. aeruginosa* (VC8263 and VC17829) were completely eradicated in our study. In addition, *P. aeruginosa* VC15184-1, a mucoid strain, and its non-mucoid derivative VC15184-2 were similarly affected by KC, indicating that a mucoid phenotype in *P. aeruginosa* is not likely to be a major factor in resistance to KC. In addition, we could not find any correlation between the mucoidy of Bcc isolates and their sensitivity toward KC suspension and its aqueous leachate (Supplementary Table S1). Noteworthy, our study revealed the high sensitivity of *P. aeruginosa* isolates toward KC and its leachate. As *P. aeruginosa* is a major opportunistic pathogen responsible for life-threatening infections, these findings will be a guide to the use of alternative metal-based antimicrobial compounds in the battle against this major recalcitrant group of pathogens. Elucidating the mode of action of KC leachates together with the potent growth inhibitory action of KC provides a specific direction to assess the therapeutic potential of KC and its derivatives for the inhibition of these major bacterial pathogens. Our studies further expand the spectrum of the activity of KC to include isolates of some of the most challenging bacterial pathogens from CF and suggest further studies of other globally important *Burkholderia* pathogens such as *B. pseudomallei* and *B. mallei*. Notably, *B. pseudomallei*, formerly known as *Pseudomonas pseudomallei* is the causative agent of melioidosis, a life-threatening infection in humans with a high fatality rate and a wide range of clinical manifestations [90,91].

To the best of our knowledge, this is the first study summarizing the antibacterial activity of a natural clay mineral and its derivatives against CF-related MDR Bcc and *P. aeruginosa* clinical pathogens. Recently, inhaled antibiotic therapy through aerosolized drug delivery has been explored as an effective method to deliver high concentrations of therapeutic agents to the lungs of patients suffering from respiratory illnesses [45,85]. Moreover, as the cornerstones of treatment for CF pulmonary infections consist of antimicrobial agents together with airway clearance therapy and treatments for affecting mucus rheology [3], KC leachate may be a potential therapeutic option, as a complementary or a suppressive antimicrobial treatment, for pathogenic colonization and chronic pulmonary infections in pwCF and in cases of CGD. Further detailed cytotoxicity investigations as well as in vivo studies in animal models remain to be carried out.

We previously characterized the geochemical and microbiological features across the KC deposit from different depths to obtain more insight into the characteristics that contribute to its antibacterial activity [64,65]. Supplementary Table S2 represents the results of the quantitative phase analysis of the KC clay sample using an X-ray diffraction method. The KC clay sample used in this study can be classified as a mixture of framework silicates and illite/chlorite type phyllosilicates, composed of silicate minerals (96.7%), mainly of tectosilicates (65.4%) known as framework silicates [64,65]. KC sample contains phyllosilicates (24.3%) including biotite, illite-type mica, and chlorite-type clinochlore [64]. Interestingly, while all the natural clay minerals with potent antibacterial activity were reported to contain smectite as the dominant mineral group [57,59,61], KC contains biotite as the major clay mineral [64,65].

We also performed elemental analyses of bulk KC samples and their aqueous leachates which characterized KC as an iron- and aluminum-rich clay mineral [64,65,66]. In addition, integrating physicochemical characterization with microbiological studies, dissected the complex antibacterial activities of KC, suggesting a multi-target mechanism of action [65,66]. Our mechanistic investigations, using *Escherichia coli*, *Staphylococcus aureus*, and *P. aeruginosa* (as representative Gram-negative, Gram-positive, and clinically important organisms, respectively), revealed that the antibacterial features of KC aqueous leachates were influenced by the presence of divalent and trivalent metal ions, in a pH-dependant manner [66]. Together these suggest a critical role for transition metal ions and aluminum in the potent antibacterial activity of KC leachates [65,66]. In fact, a low-pH buffered environment, rich in a combination of released metal ions, plays a key role and challenges treated bacteria synergistically through permeabilization of OM, destabilization of the cell membrane, and induction of oxidative stress [65]. As the OM permeability barrier is a critical contributor to Bcc antimicrobial resistance [32,38], further studies build on our current knowledge through KC active components and bacterial targets should aim to elucidate the lethal mode(s) of action of KC derivatives against Bcc and *P. aeruginosa*, particularly the role of OM. Moreover, Nunvar et al., reported recently that oxidative stress response- and transition metal metabolism-related genes in *B. cenocepacia* were affected substantially during CF chronic infections [92], so further investigations exploring these two aspects can yield more insight into the sensitivity of Bcc toward KC derivatives. Altogether, these studies can ultimately facilitate the development of more defined and consistent preparations of KC as potential suppressive or therapeutic options. Such natural mineral-based agents may offer novel weapons in our battle against MDR pathogens in the post-antibiotic era.

## Figures and Tables

**Table 1:** Resistance patterns of isolates to different classes of antimicrobial agents.

					Amikacin (10)	Gentamicin (30)	Kanamycin (30)	Neomycin (30)	Spectinomycin (100) *	Streptomycin (10)	Tobramycin (10)	Ertapenem (10)	Imipenem (10)	Meropenem (10)	Cephalothin (30)	Cephazolin (30)	Cefotetan (30)	Cefoxitin (30)	Ceftazidime (30)	Cefixime (5)	Cefpodoxime (10)	Ceftriaxone (30)	Cefotaxime (30)	Amoxicillin-clavulanic acid (30)	Ampicillin (10)	Piperacilin (100)	Colistin (10)	Polymyxin B (300)	Ciprofloxacin (5)	Levofloxacin (5)	Nalidixic acid (30)	Sulfamethoxazole- trimethoprim (25)	Sulfadiazine (250)	Trimethoprim (5)	Doxycycline (30)	Tetracycline (30)	Chloramphenicol (30)	Nitrofurantoin (300)
**No.**	**Isolate**	**Strain**	**Year**	**Source**	**Aminoglycosides**							**Carbapenems**			**1st, 2nd, 3rd Generation Cephalosporins**									**Penicillins**			**Polypeptides**		**Quinolones**			**Sulfonamides**			**Tetracyclines**			
1	*Burkholderia cepacia*	VC9490	1999	Sputum	●	●	●	●	●	●	●	●		●	●	●	●	●		●	●	●	●	●	●		●	●	●	●		●	●	●	●	●		●
2	*Burkholderia cenocepacia*	C3921 ^a^ [73,74]	1990	Sputum	●	●	●	●	●	●	●	●	●	●	●	●	●	●		●	●	●	●	●	●		●	●				●	●	●	●	●	●	●
3	*Burkholderia cenocepacia*	C8963 ^a^ [73,74]	2000	Resp ^e^	●	●	●	●	●	●	●	●		●	●	●	●	●		●				●	●		●	●	●	●	●	●	●	●	●	●	●	●
4	*Burkholderia cenocepacia*	C9343 ^a^ [73,74]	2000	Resp	●	●	●	●	●	●	●	●	●	●	●	●	●	●		●				●	●				●	●	●	●	●	●	●	●		●
5	*Burkholderia cenocepacia*	VC13195 ^b^	2006	Resp	●	●	●	●	●	●	●	●	●	●	●	●	●	●		●	●	●	●	●	●	●	●	●	●	●	●	●	●	●	●	●	●	●
6	*Burkholderia cenocepacia*	VC15185 ^b^	2010	Resp	●	●	●	●	●	●	●	●	●	●	●	●	●	●	●	●	●	●	●	●	●	●	●	●	●	●	●	●	●	●	●	●	●	●
7	*Burkholderia cenocepacia*	VC15442 ^b^	2010	Blood ^f^	●	●	●	●	●	●	●	●	●	●	●	●	●	●	●	●	●	●	●	●	●	●	●	●	●	●	●	●	●	●		●	●	●
8	*Burkholderia dolosa*	VC14902	2009	Sputum	●	●		●	●	●	●	●		●	●	●	●	●	●					●	●		●	●					●				●	●
9	*Burkholderia multivorans*	VC5602 ^c^ [75]	1993	Sputum	●	●		●	●	●	●	●		●	●	●	●	●						●	●		●	●					●					●
10	*Burkholderia multivorans*	VC16929 ^c^	2013	Sputum	●	●		●	●	●	●	●	●	●	●	●	●	●		●	●	●	●	●	●		●	●					●					●
11	*Burkholderia stabilis*	VC7909	1993	Sputum					●	●		●		●	●	●	●	●	●	●	●	●	●	●	●		●		●	●	●	●	●	●	●	●	●	●
12	*Burkholderia vietnamiensis*	VC9237 [76]	1998	Resp						●		●		●	●	●	●	●		●				●	●		●	●			●	●					●	●
13	*Pseudomonas aeruginosa*	VC8263 [68]	1997	Resp								●		●	●	●	●	●		●	●			●	●					●	●	●	●	●	●		●	●
14	*Pseudomonas aeruginosa*	VC15184-1 ^d^	2010	Resp			●	●	●	●		●		●	●	●	●	●		●	●	●	●	●	●						●	●		●	●	●	●	●
15	*Pseudomonas aeruginosa*	VC15184-2 ^d^	2010	Resp			●	●	●	●		●		●	●	●	●	●		●	●	●	●	●	●						●	●		●	●	●	●	●
16	*Pseudomonas aeruginosa*	VC17829 [68]	2015	Sputum			●	●	●	●		●	●	●	●	●	●	●	●	●	●	●	●	●	●							●	●	●				●
17	*Stenotrophomonas maltophilia*	VC13512	2006	Sputum	●	●	●	●	●	●	●	●	●	●	●	●	●	●	●	●	●	●	●	●	●	●							●	●				●

Filled black circles indicate resistance (inhibition ≤ 2 mm from the edge of the disks of antibiotic); no mark indicates the wider zone of inhibition (at least three replicates for each antibiotic). The amount (micrograms) per disk of antibiotics (Oxoid, BBL) is indicated in parentheses. * Spectinomycin is an aminocyclitol antibiotic, but as it is structurally related to the aminoglycosides, often considered alongside this group of antibiotics Refs [77,78]. ^a,b,c^ sequential isolates from the same patients that were previously evaluated for strain type by random amplified polymorphic DNA (RAPD) analysis Ref [78]. ^d^ two morphotypes of the strain VC15184: (−1, mucoid, and −2, non-mucoid strain which was recovered during the course of this study and confirmed to have the same RAPD phenotype). The remaining strains were independent isolates from one patient. ^e^ Resp indicates a sample from the respiratory tract, either sputum or throat/cough swab. ^f^ This strain was isolated from a patient’s blood sample during “cepacia” syndrome.

## Data Availability

Data are presented in the paper as well as the supporting information.

## Supplementary Materials

The following are available online at https://www.mdpi.com/article/10.3390/microorganisms11010150/s1, Table S1. Mucoidy phenotype of the collection of CF isolates, Text S1. Mineralogical composition of Kisameet clay (KC) by X-ray diffraction, Table S2. Mineralogical composition of KC clay mineral using X-ray diffraction. Reference [93] is cited in the supplementary materials.
