# Supplementary material for: Antibacterial Activity of a Natural Clay Mineral against Burkholderia cepacia Complex and Other Bacterial Pathogens Isolated from People with Cystic Fibrosis
Source: Microorganisms. 2023 Jan 6;11(1):150. doi: 10.3390/microorganisms11010150 (PMC9862493; doi:10.3390/microorganisms11010150)
Supplement: Supplementary file 1 [file microorganisms-11-00150-s001.zip › microorganisms-2015576-supplementary.pdf]

# SUPPLEMENTAL MATERIAL

*Article*

# Antibacterial Activity of a Natural Clay Mineral against *Burkholderia cepacia* Complex and Other Bacterial Pathogens Isolated from People with Cystic Fibrosis

**Shekooh Behroozian [1,*], James E. A. Zlosnik [2,†], Wanjing Xu [3], Loretta Y. Li [3] and Julian E. Davies [4,*]**

[1] Department of Chemical and Biological Engineering, University of British Columbia, 2360 E Mall, Vancouver, BC V6T 1Z3, Canada
[2] Centre for Understanding and Preventing Infection in Children, Division of Infectious Diseases, Department of Pediatrics, BC Children's Hospital Research Institute, University of British Columbia, Vancouver, BC V5Z 4H4, Canada
[3] Department of Civil Engineering, University of British Columbia, 6250 Applied Science Ln, Vancouver, BC V6T 1Z3, Canada
[4] Department of Microbiology and Immunology, University of British Columbia, 2350 Health Sciences Mall, Vancouver, BC V6T 1Z3, Canada
* Correspondence: shekooh@mail.ubc.ca (S.B.); jed@mail.ubc.ca (J.E.D.)
† Current address: BC Centre for Disease Control, Provincial Health Services Authority, BC and Department of Pathology and Laboratory Medicine, Faculty of Medicine, University of British Columbia, Vancouver, BC V5Z 4R4, Canada

**Table S1.** Mucoidy phenotypes of the collection of CF isolates.

| No. | Isolate | Strain | Genomovar | Isolation Date | Source | Mucoidy | Reference |
|---|---|---|---|---|---|---|---|
| 1 | *Burkholderia cepacia* | VC9490 | I | 02/1999 | Sputum | 1+ | |
| 2 | *Burkholderia cenocepacia* | C3921[a] | III | 10/1990 | Sputum | 1+ | [1,2] |
| 3 | *Burkholderia cenocepacia* | C8963[a] | III | 01/2000 | Resp[d] | 1+ | [1,2] |
| 4 | *Burkholderia cenocepacia* | C9343[a] | III | 10/2000 | Resp | 3+ | [1,2] |
| 5 | *Burkholderia cenocepacia* | VC13195[b] | III | 01/2006 | Resp | non-mucoid | |
| 6 | *Burkholderia cenocepacia* | VC15185[b] | III | 04/2010 | Resp | 1+ | |
| 7 | *Burkholderia cenocepacia* | VC15442[b] | III | 12/2010 | Blood | non-mucoid | |
| 8 | *Burkholderia dolosa* | VC14902 | VI | 09/2009 | Sputum | 1+ | |
| 9 | *Burkholderia multivorans* | VC5602[c] | II | 11/1993 | Sputum | non-mucoid | [3] |
| 10 | *Burkholderia multivorans* | VC16929[c] | II | 11/2013 | Sputum | 1+ | |
| 11 | *Burkholderia stabilis* | VC7909 | IV | 10/1993 | Sputum | non-mucoid | |
| 12 | *Burkholderia vietnamiensis* | VC9237 | V | 11/1998 | Resp | 1+ | [4] |
| 13 | *Pseudomonas aeruginosa* | VC8263 | - | 06/1997 | Resp | non-mucoid | [5] |
| 14 | *Pseudomonas aeruginosa* | VC15184-1 | - | 04/2010 | Resp | 4+ | |
| 15 | *Pseudomonas aeruginosa* | VC15184-2 | - | 04/2010 | Resp | non-mucoid | |
| 16 | *Pseudomonas aeruginosa* | VC17829 | - | 07/2015 | Sputum | non-mucoid | [5] |
| 17 | *Stenotrophomonas maltophilia* | VC13512 | - | 10/2006 | Sputum | 1+ | |

[a], [b], [c] sequential isolates from the same patients that were previously evaluated for strain type by random amplified polymorphic DNA analysis [Ref 6].
[d] Resp indicates a sample from the respiratory tract, either sputum or throat/cough swab.
Mucoidy was assessed on Lysogeny (Miller) (LB) agar plates containing 1.5 % (*w*/*v*) agar and incubated for 48 h at 37 °C. Mucoidy was reported as non-mucoid isolates showing no mucoidy or mucoid isolates with scores of 1+ to 4+ depending on the extent of mucoidy exhibition.

**Text S1.** Mineralogical composition of Kisameet clay (KC) by X-ray diffraction

The quantitative mineralogical profile of the KC clay sample was analyzed by Rietveld refinements using the X-ray diffraction method as described previously [Ref 7-9]. Briefly, the clay sample was reduced to the optimum grain-size range ( less than 10 μm) for the quantitative X-ray analysis. This was performed through grinding under ethanol using a vibratory McCrone Micronising Mill for 7 min. Continuous-scan X-ray powder-diffraction data was obtained over a range 3-80° 2-θ range with step-size increments of 0.03° 2- θ and a counting time of 0.7 seconds per step, with CoKalpha radiation on a Bruker D8 Focus Bragg-Brentano diffractometer equipped as well as a Fe monochromator foil, 0.6 mm (0.3°) divergence slit, incident- and diffracted-beam Soller slits, and a LynxEye detector. Then the long fine-focus Co X-ray tube was operated at 40 mA and 35 kV, using a take-off angle of 6°. The X-ray diffractogram was analyzed and characterized using the International Centre for Diffraction Database PDF-4 and Search-Match software by Bruker. X-ray powder-diffraction data of the clay sample was refined using the Rietveld program Topas 4.2 (Bruker AXS).

**Table S2.** Mineralogical composition of KC clay mineral using X-ray diffraction method

| Mineral | Classification | Ideal Formula | KC35 |
|---|---|---|---|
| Actinolite | Inosilicate | $Ca_2(Mg,Fe^{2+})_5Si_8O_{22}(OH)_2$ | 9.3 |
| Albite low, calcian | Tectosilicate | $NaAlSi_3O_8$ | 40.8 |
| Biotite 1M | Phyllosilicate | $K(Mg,Fe)_3AlSi_3O_{10}(OH)_2$ | 10 |
| Calcite | Calcium carbonate | $CaCO_3$ | nd* |
| Clinochlore II | Phyllosilicate | $(Mg,Fe^{2+})_5Al(Si_3Al)O_{10}(OH)_8$ | 9 |
| Goethite | Iron-bearing hydroxide | FeO(OH) | nd |
| Gypsum | Soft sulphate | $CaSO_4 \cdot 2H_2O$ | 0.9 |
| Illite-Muscovite | Phyllosilicate | $K_{0.65}Al_{2.0}Al_{0.65}Si_{3.35}O_{10}(OH)_2$ | 5.3 |
| Laumontite | Tectosilicate | $CaAl_2Si_4O_{12} \cdot 4H_2O$ | 3.5 |
| Magnetite | Iron oxide | $Fe_3O_4$ | nd |
| Microcline ordered | Tectosilicate | $KAlSi_3O_8$ | 8.9 |
| Quartz low | Tectosilicate | $SiO_2$ | 12.2 |
| Total tectosilicates | | | 65.4 |
| Total phyllosilicates | | | 24.3 |
| Total inosilicate | | | 9.3 |
| Total silicates | | | 96.7 |
| Total | | | 100 |

* not detected

## References


1. Miller, R.R.; Hird, T.J.; Tang, P.; Zlosnik, J.E. Whole-Genome Sequencing of Three Clonal Clinical Isolates of *B. cenocepacia* from a Patient with Cystic Fibrosis. *PloS one*. **2015**, *10*(11), e0143472, https://doi.org/10.1371/journal.pone.0143472
2. Zlosnik, J.E.; Speert, D.P. The role of mucoidy in virulence of bacteria from the *Burkholderia cepacia* complex: a systematic proteomic and transcriptomic analysis. *J. Infect. Dis.* **2010**, *202*(5), 770–781, https://doi.org/10.1086/655663
3. Silva, I.N.; Santos, P.M.; Santos, M.R.; Zlosnik, J.E.; Speert, D.P.; Buskirk, S.W.; Bruger, E.L.; Waters, C.M.; Cooper, V.S.; Moreira, L.M. Long-Term Evolution of *Burkholderia multivorans* during a Chronic Cystic Fibrosis Infection Reveals Shifting Forces of Selection. *mSystems*. **2016**, *1*(3), e00029-16, https://doi.org/10.1128/mSystems.00029-16
4. Kennedy, S.; Beaudoin, T.; Yau, Y.C.; Caraher, E.; Zlosnik, J.E.; Speert, D.P.; LiPuma, J.J.; Tullis, E.; Waters, V. Activity of Tobramycin against Cystic Fibrosis Isolates of *Burkholderia cepacia* Complex Grown as Biofilms. *Antimicrob. Agents Chemother.* **2016**, *60*(1), 348–355, https://doi.org/10.1128/AAC.02068-15
5. Speert, D.P.; Campbell, M.E.; Henry, D.A.; Milner, R.; Taha, F.; Gravelle, A.; Davidson, A.G.; Wong, L.T.; Mahenthiralingam, E. Epidemiology of *Pseudomonas aeruginosa* in cystic fibrosis in British Columbia, Canada. *Am. J. Respir. Crit. Care Med.* **2002**, *166*(7), 988–993, https://doi.org/10.1164/rccm.2203011
6. Speert, D.P.; Henry, D.; Vandamme, P.; Corey, M.; Mahenthiralingam, E. Epidemiology of *Burkholderia cepacia* complex in patients with cystic fibrosis, Canada. *Emerg. Infect. Dis.* **2002**, *8*(2), 181–187, https://doi.org/10.3201/eid0802.010163
7. Bish, D.L. Studies of clays and clay minerals using x-ray powder diffraction and the Rietveld method. **1993**. United States. Available from: http://www.osti.gov/servlets/purl/10192067. (accessed on 26 June 2015).
8. Svensson, S.L., Behroozian, S., Xu, W., Surette, M.G., Li, L., Davies, J. Kisameet Glacial Clay: an Unexpected Source of Bacterial Diversity. *mBio*, **2017**, *8*(3), e00590-17, https://doi.org/10.1128/mBio.00590-17
9. Behroozian, S. Antimicrobial properties of Kisameet clay, a natural clay mineral from British Columbia, Canada. Doctoral thesis., University of British Columbia, Vancouver, B.C., Canada, **2019**. Available on: http://open.library.ubc.ca/collections/ubctheses/24/items/1.0380811. (accessed on 4 September 2019).
